# 2,2,2-Trifluoro­ethyl 4-methyl­benzene­sulfonate

**DOI:** 10.1107/S1600536810038894

**Published:** 2010-10-09

**Authors:** Song Xia, Ya-Bin Shi, Fei-Fei He, Hai-Bo Wang

**Affiliations:** a College of Food Science and Light Industry, Nanjing University of Technology, Xinmofan Road No. 5 Nanjing, Nanjing 210009, People’s Republic of China; b College of Science, Nanjing University of Technology, Xinmofan Road No. 5 Nanjing, Nanjing 210009, People’s People’s Republic of China

## Abstract

In the crystal structure of the title compound, C_9_H_9_F_3_O_3_S, inter­molecular C—H⋯O hydrogen bonds link the mol­ecules along the *c-*axis direction. Also present are slipped π–π stacking inter­actions between phenyl­ene rings, with perpendicular inter­planar distances of 3.55 (2) Å and centroid–centroid distances of 3.851 (2) Å.

## Related literature

The title compound is a reactive electrophile and a useful inter­mediate in organic synthesis. For general background and the synthesis, see: Gøgsig *et al.* (2008 ▶). For a similar structure, see: Asano *et al.* (2009 ▶). For bond-length data, see: Allen *et al.* (1987 ▶).
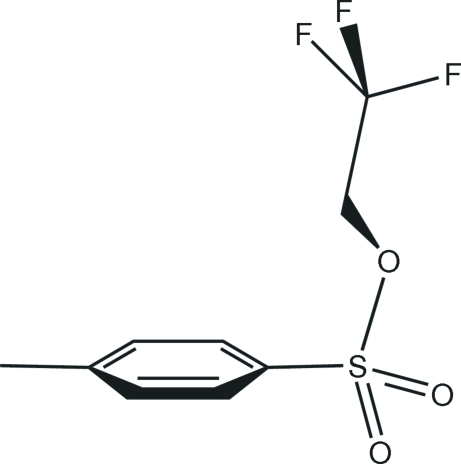


## Experimental

### 

#### Crystal data

C_9_H_9_F_3_O_3_S
*M* *_r_* = 254.22
Monoclinic, 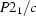

*a* = 8.3760 (17) Å
*b* = 11.827 (2) Å
*c* = 11.145 (2) Å
β = 94.54 (3)°
*V* = 1100.6 (4) Å^3^
*Z* = 4
Mo *K*α radiation
μ = 0.33 mm^−1^
*T* = 293 K
0.30 × 0.10 × 0.10 mm

#### Data collection

Enraf–Nonius CAD-4 diffractometer
Absorption correction: ψ scan (North *et al.*, 1968 ▶) *T*_min_ = 0.909, *T*_max_ = 0.968
2149 measured reflections
2005 independent reflections
1355 reflections with *I* > 2σ(*I*)
*R*_int_ = 0.012
3 standard reflections every 200 reflections  intensity decay: 1%

#### Refinement

*R*[*F* ^2^ > 2σ(*F* ^2^)] = 0.057
*wR*(*F* ^2^) = 0.172
*S* = 1.00
2005 reflections
146 parameters
H-atom parameters constrained
Δρ_max_ = 0.21 e Å^−3^
Δρ_min_ = −0.29 e Å^−3^

### 

Data collection: *CAD-4 EXPRESS* (Enraf–Nonius, 1989 ▶); cell refinement: *CAD-4 EXPRESS*; data reduction: *XCAD4* (Harms & Wocadlo, 1995 ▶); program(s) used to solve structure: *SHELXS97* (Sheldrick, 2008 ▶); program(s) used to refine structure: *SHELXL97* (Sheldrick, 2008 ▶); molecular graphics: *SHELXTL* (Sheldrick, 2008 ▶); software used to prepare material for publication: *PLATON* (Spek, 2009 ▶).

## Supplementary Material

Crystal structure: contains datablocks global, I. DOI: 10.1107/S1600536810038894/zl2306sup1.cif

Structure factors: contains datablocks I. DOI: 10.1107/S1600536810038894/zl2306Isup2.hkl

Additional supplementary materials:  crystallographic information; 3D view; checkCIF report

## Figures and Tables

**Table 1:** Hydrogen-bond geometry (Å, °)

*D*—H⋯*A*	*D*—H	H⋯*A*	*D*⋯*A*	*D*—H⋯*A*
C8—H8*B*⋯O1^i^	0.97	2.51	3.225 (4)	131

Symmetry code: (i) 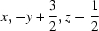.

**Fig. 1.**
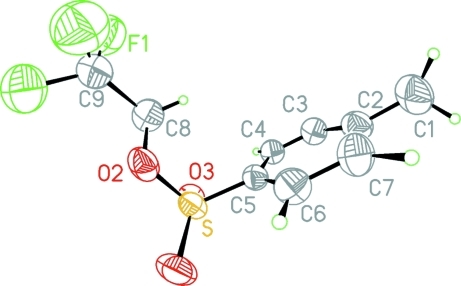
The molecular structure of the title molecule, with the atom-numbering scheme. Displacement ellipsoids are drawn at the 50% probability level.

**Fig. 2.**
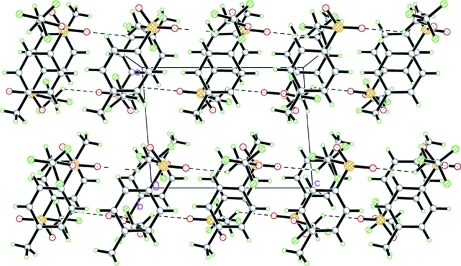
A packing diagram of 2,2,2-trifluoroethyl 4-methylbenzenesulfonate. Dashed lines indicate intermolecular C-H···O interactions.

## supplementary crystallographic information

### Comment

Electrophilic reagents play an important role in the synthesis of organic compounds and are often used in the synthesis of organic intermediates. (Asano *et al.* 2009). The title compound, 2,2,2-trifluoroethyl 4-methylbenzenesulfonate, is a electrophilic vinylation reagent commonly used for the synthesis of compounds such as vinyl styrenes that in turn find use as valuable intermediates in the synthesis of fine chemicals and as precursors to functionalized polymers (Gøgsig *et al.*, 2008).

We report here in the crystal structure of the title compound, 2,2,2-trifluoroethyl 4-methylbenzenesulfonate. In the molecule of the title compound (Fig. 1), bond lengths (Allen *et al.*, 1987) and angles are within normal ranges. An intramolecular C-H···O hydrogen bond (Table 1) results in the formation of a five-membered ring (C4/C5/S/O3/H4A). In the crystal structure, the weak intermolecular C8-H8···O1 hydrogen bond connects the molecules along the direction of the *c* axis (Fig. 2). Also present are slipped π-π stacking interactions between phenylene rings with perpendicular interplanar distances of 3.55 (2) Å and centroid to centroid distances of 3.851 (2) Å (symmetry operator for the second molecule: -x, 2-y, -z).

### Experimental

The title compound, 2,2,2-trifluoroethyl 4-methylbenzenesulfonate was prepared on a literature procedure (Gøgsig *et al.*, 2008). 2,2,2-Trifluoroethanol (19.90 mmol) and triethylamine (71.70 mmol) were dissolved in dry dichloromethane (20.0 mL). The solution was cooled to 273K and tosyl chloride (24.9 mmol) was added. The reaction was stirred at 273K for 1 h before being allowed to warm to room temperature. Hereafter the reaction was stirred at room temperature overnight. The organic phase was washed with brine (2 × 50 mL) and dried over sodium sulfate. After concentration in vacuo the crude product was purified by flash cromatography on silica gel using pentane/dichloromethane (4:1) and pentane/dichloromethane (3:1) as the eluents. This afforded of the title compound (96 % yield) as a colorless solid. Crystals suitable for X-ray analysis were obtained by slow evaporation of a methanol solution.

### Refinement

H atoms were positioned geometrically, with  C—H = 0.93, 0.98 and 0.96 Å for aromatic, methylene and methyl H, respectively, and constrained to ride on their parent atoms, with U_iso_(H) = xU_eq_(C), where x = 1.5 for methyl H and x = 1.2 for all other H atoms.

### Crystal data

**Table:**

C_9_H_9_F_3_O_3_S	*F*(000) = 520
*M**_r_* = 254.22	*D*_x_ = 1.534 Mg m^−^^3^
Monoclinic, *P*2_1_/*c*	Melting point: 312 K
Hall symbol: -P 2ybc	Mo *K*α radiation, λ = 0.71073 Å
*a* = 8.3760 (17) Å	Cell parameters from 25 reflections
*b* = 11.827 (2) Å	θ = 9–13°
*c* = 11.145 (2) Å	µ = 0.33 mm^−^^1^
β = 94.54 (3)°	*T* = 293 K
*V* = 1100.6 (4) Å^3^	Needle, colourless
*Z* = 4	0.30 × 0.10 × 0.10 mm

### Data collection

**Table:**

Enraf–Nonius CAD-4 diffractometer	1355 reflections with *I* > 2σ(*I*)
Radiation source: fine-focus sealed tube	*R*_int_ = 0.012
graphite	θ_max_ = 25.3°, θ_min_ = 2.4°
ω/2θ scans	*h* = 0→10
Absorption correction: ψ scan (North *et al.*, 1968)	*k* = 0→14
*T*_min_ = 0.909, *T*_max_ = 0.968	*l* = −13→13
2149 measured reflections	3 standard reflections every 200 reflections
2005 independent reflections	intensity decay: 1%

### Refinement

**Table:**

Refinement on *F*^2^	Primary atom site location: structure-invariant direct methods
Least-squares matrix: full	Secondary atom site location: difference Fourier map
*R*[*F*^2^ > 2σ(*F*^2^)] = 0.057	Hydrogen site location: inferred from neighbouring sites
*wR*(*F*^2^) = 0.172	H-atom parameters constrained
*S* = 1.00	*w* = 1/[σ^2^(*F*_o_^2^) + (0.1*P*)^2^ + 0.350*P*] where *P* = (*F*_o_^2^ + 2*F*_c_^2^)/3
2005 reflections	(Δ/σ)_max_ < 0.001
146 parameters	Δρ_max_ = 0.21 e Å^−^^3^
0 restraints	Δρ_min_ = −0.29 e Å^−^^3^

### Special details

**Table:**

Geometry. All esds (except the esd in the dihedral angle between two l.s. planes) are estimated using the full covariance matrix. The cell esds are taken into account individually in the estimation of esds in distances, angles and torsion angles; correlations between esds in cell parameters are only used when they are defined by crystal symmetry. An approximate (isotropic) treatment of cell esds is used for estimating esds involving l.s. planes.
Refinement. Refinement of F^2^ against ALL reflections. The weighted R-factor wR and goodness of fit S are based on F^2^, conventional R-factors R are based on F, with F set to zero for negative F^2^. The threshold expression of F^2^ > 2sigma(F^2^) is used only for calculating R-factors(gt) etc. and is not relevant to the choice of reflections for refinement. R-factors based on F^2^ are statistically about twice as large as those based on F, and R- factors based on ALL data will be even larger.

### Fractional atomic coordinates and isotropic or equivalent isotropic displacement parameters (Å^2^)

**Table:**

	*x*	*y*	*z*	*U*_iso_*/*U*_eq_	
S	0.22668 (10)	0.81228 (8)	0.14051 (7)	0.0524 (3)	
O1	0.2130 (3)	0.8490 (2)	0.2610 (2)	0.0726 (8)	
O2	0.2305 (3)	0.6787 (2)	0.15228 (18)	0.0564 (7)	
O3	0.3590 (3)	0.8450 (2)	0.0778 (2)	0.0685 (8)	
C1	−0.3915 (5)	0.8728 (4)	−0.1679 (4)	0.0878 (14)	
H1B	−0.3695	0.8862	−0.2499	0.132*	
H1C	−0.4531	0.9345	−0.1399	0.132*	
H1D	−0.4509	0.8038	−0.1630	0.132*	
C2	−0.2361 (5)	0.8634 (3)	−0.0908 (3)	0.0590 (10)	
C3	−0.0892 (5)	0.8731 (3)	−0.1392 (3)	0.0569 (9)	
H3B	−0.0870	0.8893	−0.2207	0.068*	
C4	0.0537 (4)	0.8594 (3)	−0.0705 (3)	0.0511 (8)	
H4A	0.1509	0.8650	−0.1051	0.061*	
C5	0.0493 (4)	0.8369 (3)	0.0522 (3)	0.0442 (8)	
C6	−0.0958 (4)	0.8308 (3)	0.1034 (3)	0.0517 (9)	
H6A	−0.0981	0.8178	0.1856	0.062*	
C7	−0.2343 (5)	0.8439 (3)	0.0336 (3)	0.0623 (10)	
H7A	−0.3310	0.8399	0.0690	0.075*	
C8	0.2628 (5)	0.6142 (3)	0.0482 (3)	0.0662 (11)	
H8A	0.3679	0.6333	0.0234	0.079*	
H8B	0.1840	0.6316	−0.0178	0.079*	
C9	0.2565 (6)	0.4960 (4)	0.0770 (4)	0.0717 (11)	
F3	0.1153 (4)	0.4632 (3)	0.1101 (3)	0.1040 (9)	
F2	0.3549 (4)	0.4617 (2)	0.1673 (3)	0.1064 (10)	
F1	0.2812 (4)	0.4335 (3)	−0.0197 (3)	0.1119 (10)	

### Atomic displacement parameters (Å^2^)

**Table:**

	*U*^11^	*U*^22^	*U*^33^	*U*^12^	*U*^13^	*U*^23^
S	0.0482 (5)	0.0686 (6)	0.0399 (5)	0.0007 (4)	0.0000 (3)	−0.0052 (4)
O1	0.0794 (19)	0.095 (2)	0.0415 (14)	0.0052 (15)	−0.0036 (12)	−0.0168 (14)
O2	0.0625 (16)	0.0732 (17)	0.0339 (12)	0.0100 (12)	0.0057 (10)	0.0013 (11)
O3	0.0511 (15)	0.0831 (19)	0.0722 (17)	−0.0068 (13)	0.0115 (13)	0.0018 (15)
C1	0.075 (3)	0.096 (3)	0.087 (3)	0.005 (3)	−0.027 (2)	0.007 (3)
C2	0.063 (2)	0.056 (2)	0.055 (2)	0.0001 (18)	−0.0071 (18)	−0.0006 (18)
C3	0.070 (2)	0.070 (2)	0.0309 (17)	0.0066 (19)	0.0043 (16)	0.0094 (16)
C4	0.054 (2)	0.061 (2)	0.0397 (18)	0.0017 (16)	0.0090 (15)	0.0023 (16)
C5	0.0430 (18)	0.0482 (18)	0.0411 (17)	0.0026 (14)	0.0024 (13)	−0.0028 (14)
C6	0.051 (2)	0.063 (2)	0.0421 (18)	−0.0018 (16)	0.0120 (15)	0.0010 (16)
C7	0.052 (2)	0.069 (2)	0.066 (2)	−0.0010 (18)	0.0102 (18)	0.005 (2)
C8	0.079 (3)	0.070 (3)	0.052 (2)	0.007 (2)	0.0204 (19)	−0.0014 (19)
C9	0.079 (3)	0.068 (3)	0.067 (3)	−0.003 (2)	−0.005 (2)	−0.004 (2)
F3	0.096 (2)	0.110 (2)	0.106 (2)	−0.0209 (17)	0.0106 (16)	0.0062 (17)
F2	0.106 (2)	0.094 (2)	0.116 (2)	0.0091 (16)	−0.0117 (18)	0.0086 (17)
F1	0.132 (3)	0.096 (2)	0.109 (2)	0.0005 (18)	0.0163 (19)	−0.0149 (17)

### Geometric parameters (Å, °)

**Table:**

S—O3	1.410 (3)	C4—C5	1.397 (4)
S—O1	1.425 (2)	C4—H4A	0.9300
S—O2	1.585 (3)	C5—C6	1.385 (4)
S—C5	1.740 (3)	C6—C7	1.354 (5)
O2—C8	1.432 (4)	C6—H6A	0.9300
C1—C2	1.506 (5)	C7—H7A	0.9300
C1—H1B	0.9600	C8—C9	1.437 (6)
C1—H1C	0.9600	C8—H8A	0.9700
C1—H1D	0.9600	C8—H8B	0.9700
C2—C3	1.387 (5)	C9—F2	1.314 (5)
C2—C7	1.404 (5)	C9—F3	1.325 (5)
C3—C4	1.379 (5)	C9—F1	1.336 (5)
C3—H3B	0.9300		
			
O3—S—O1	120.54 (18)	C6—C5—C4	120.4 (3)
O3—S—O2	107.64 (15)	C6—C5—S	119.7 (2)
O1—S—O2	103.21 (15)	C4—C5—S	119.9 (3)
O3—S—C5	110.08 (16)	C7—C6—C5	119.8 (3)
O1—S—C5	110.68 (16)	C7—C6—H6A	120.1
O2—S—C5	102.95 (15)	C5—C6—H6A	120.1
C8—O2—S	117.9 (2)	C6—C7—C2	121.9 (3)
C2—C1—H1B	109.5	C6—C7—H7A	119.1
C2—C1—H1C	109.5	C2—C7—H7A	119.1
H1B—C1—H1C	109.5	O2—C8—C9	109.0 (3)
C2—C1—H1D	109.5	O2—C8—H8A	109.9
H1B—C1—H1D	109.5	C9—C8—H8A	109.9
H1C—C1—H1D	109.5	O2—C8—H8B	109.9
C3—C2—C7	117.2 (3)	C9—C8—H8B	109.9
C3—C2—C1	121.7 (4)	H8A—C8—H8B	108.3
C7—C2—C1	121.1 (4)	F2—C9—F3	102.5 (4)
C4—C3—C2	122.1 (3)	F2—C9—F1	108.7 (4)
C4—C3—H3B	118.9	F3—C9—F1	105.1 (4)
C2—C3—H3B	118.9	F2—C9—C8	116.1 (4)
C3—C4—C5	118.5 (3)	F3—C9—C8	113.4 (4)
C3—C4—H4A	120.7	F1—C9—C8	110.4 (4)
C5—C4—H4A	120.7		
			
O3—S—O2—C8	−44.3 (3)	O1—S—C5—C4	149.6 (3)
O1—S—O2—C8	−172.8 (3)	O2—S—C5—C4	−100.7 (3)
C5—S—O2—C8	72.0 (3)	C4—C5—C6—C7	1.7 (5)
C7—C2—C3—C4	2.8 (6)	S—C5—C6—C7	−176.5 (3)
C1—C2—C3—C4	−176.9 (4)	C5—C6—C7—C2	0.2 (6)
C2—C3—C4—C5	−1.1 (6)	C3—C2—C7—C6	−2.4 (6)
C3—C4—C5—C6	−1.2 (5)	C1—C2—C7—C6	177.4 (4)
C3—C4—C5—S	176.9 (3)	S—O2—C8—C9	−178.5 (3)
O3—S—C5—C6	−168.1 (3)	O2—C8—C9—F2	−57.8 (5)
O1—S—C5—C6	−32.3 (3)	O2—C8—C9—F3	60.4 (5)
O2—S—C5—C6	77.4 (3)	O2—C8—C9—F1	178.0 (3)
O3—S—C5—C4	13.8 (3)		

### Hydrogen-bond geometry (Å, °)

**Table:**

*D*—H···*A*	*D*—H	H···*A*	*D*···*A*	*D*—H···*A*
C4—H4A···O3	0.93	2.59	2.938 (4)	103
C8—H8B···O1^i^	0.97	2.51	3.225 (4)	131

Symmetry codes: (i) *x*, −*y*+3/2, *z*−1/2.
